# Implicit learning of non-verbal regularities by deaf children with cochlear implants: An investigation with a dynamic temporal prediction task

**DOI:** 10.1371/journal.pone.0251050

**Published:** 2021-05-12

**Authors:** Ambra Fastelli, Giovanni Mento, Chloë Ruth Marshall, Barbara Arfé

**Affiliations:** 1 Department of Developmental Psychology and Socialisation, University of Padua, Padova, Italy; 2 Bruno Kessler Foundation, Trento, Italy; 3 Department of General Psychology, University of Padua, Padova, Italy; 4 UCL Institute of Education, University College London, London, United Kingdom; 5 University of Padua, Centre for Hearing, Speech, and Music research in Venice, Venice, Italy; University of Trento, ITALY

## Abstract

Some deaf children continue to show difficulties in spoken language learning after cochlear implantation. Part of this variability has been attributed to poor implicit learning skills. However, the involvement of other processes (e.g. verbal rehearsal) has been underestimated in studies that show implicit learning deficits in the deaf population. In this study, we investigated the relationship between auditory deprivation and implicit learning of temporal regularities with a novel task specifically designed to limit the load on working memory, the amount of information processing, and the visual-motor integration skills required. Seventeen deaf children with cochlear implants and eighteen typically hearing children aged 5 to 11 years participated. Our results revealed comparable implicit learning skills between the two groups, suggesting that implicit learning might be resilient to a lack of early auditory stimulation. No significant correlation was found between implicit learning and language tasks. However, deaf children’s performance suggests some weaknesses in inhibitory control.

## Introduction

Approximately half of cochlear-implanted deaf children (estimates ranging between 81% for speech production to 43% for receptive vocabulary) show poorer oral language performance compared with their typically hearing peers around the age when they enter primary school [1]. Moreover, deaf children with cochlear implants vary greatly in their language and learning outcomes [e.g., 2–5]. Early diagnosis, the age of implantation, the type of auditory compensation (e.g. having a bilateral rather than a unilateral cochlear implant), and the prolonged use of the hearing device are among the main factors that contribute to explaining this variability [5–8]. Recent progress in early diagnosis (e.g., neonatal hearing screening) and in prosthetic technology is helping to narrow the gap between the linguistic performance of deaf children with cochlear implants and children with typical levels of hearing [5, 9, 10]. However, having better access to spoken language does not represent a comprehensive solution for the problems that children with prelingual deafness experience in language and literacy learning [11]. Other important factors include the frequency of speech and language therapy, the socio-economic status of the family, the child’s birth order, parental involvement in children’s learning activities, and the time spent reading books [3–5]. Nevertheless, the academic outcomes obtained by deaf children with cochlear implants at 8 years of age are only partly explained by these factors, even when combined with intelligence (IQ) scores: a substantial proportion of the variance (between 31 and 65%) remains unexplained [5].

Investigating the factors that may explain this variability has become a research priority. Studies in this field can contribute to explaining individual differences in language outcomes following cochlear implantation. Also, having a better understanding of how deaf children with implants learn will support the development of instructional methods that match their strengths and needs. And finally, but no less important, the knowledge obtained may help with the early identification of young deaf children who may be at risk for poor language outcomes following cochlear implantation.

Auditory stimulation supports oral language learning through the development of phonological processing skills and phonemic awareness [12]. Some researchers have hypothesized that it could also have a broader role, scaffolding general implicit learning processes. Implicit learning is considered to be a domain-general ability that allows implicit detection and elaboration of distributional statistical regularities that are recurrent in inbound information regardless of input modality [13]. It occurs incidentally, without intention, and in a manner that is opaque to explanation [14]. It plays a crucial role during the early stages of language development [15, 16], and is considered a fundamental mechanism for human development and everyday life [17]. According to the hypothesis known as "auditory scaffolding”, early access to sound has a key role in bootstrapping the domain-general neurocognitive systems responsible for encoding, maintaining and retrieving temporal or sequential information from the environment [18]. Hence, early auditory deprivation may have widespread effects on the way these neurocognitive networks develop, with long-term effects on the capacity to process information that might go beyond the auditory system [19], thereby explaining some of the individual variability in linguistic outcomes within deaf children [19, 20]. Experimental evidence in favour of this hypothesis comes from several studies that found impaired sequence learning in deaf children with cochlear implants [20, 21] and in deaf adults who use hearing devices (i.e. either hearing aids or cochlear implants) [22]. The authors have interpreted these results as evidence that hearing experience is important for the development of implicit temporal-sequence learning skills.

In contrast, other studies have found that deaf individuals do *not* differ from typically hearing people in their implicit learning of regularities [23–27]. The findings from these studies challenge the Auditory Scaffolding Hypothesis and highlight at least two relevant issues: (1) for studies supporting the Auditory Scaffolding Hypothesis, findings of an implicit learning impairment cannot be unequivocally attributed to auditory deprivation; (2) given that not all studies have found an implicit learning impairment in participants with hearing loss, it is essential to investigate the cause(s) of these contradictory results.

It is very hard to distinctly attribute the cause of the results to auditory deprivation alone when participants experienced various degrees of language deprivation along with their auditory deprivation. This point has been investigated by Hall et al. [25] who found that both deaf and typically hearing children show robust implicit sequence learning regardless of having experienced auditory or language deprivation. The authors also noted that both deaf signing children with signing parents (who are auditory deprived but not language deprived) and deaf non-signing children with hearing non-signing parents (who are auditory deprived *and* language deprived) were successful in the implicit learning task. The authors, however, raised doubts about tasks that are widely used to assess implicit learning, finding significant results only with a task based on reaction times. Therefore, it is essential to investigate the cause(s) of these contradictory results and to consider the possible contribution of other cognitive factors during the implicit learning assessment. For example, the differences in implicit learning between deaf and typically hearing participants may reflect a confound with explicit learning factors, such as verbal recoding and verbal rehearsal [23, 27], which are less efficient in the deaf population [28].

Most of the implicit learning studies using unfamiliar stimuli that could not be easily labelled found no differences in learning between typically hearing children and deaf children [27], and found that variability in deaf children’s performance is better explained by age and explicit learning skills [23]. An exception is a study by Gremp et al. [21], who investigated the possible interaction effect of input nameability (i.e. the ease with which an input could be verbally recoded and labelled) and sequential processing in deaf children and typically hearing children using an experimental computer-based task. The group composed of deaf children performed worse than the typically hearing group regardless of the condition (easily-nameable vs. difficult-to-name stimuli). The authors interpreted these results as supporting the Auditory Scaffolding Hypothesis. However, again, other important factors may have been neglected. In particular, one-third of the deaf participants in this study were reported to have an additional diagnosis of Attention-Deficit/Hyperactivity Disorder. Since the three primary characteristics of this condition are inattention, hyperactivity, and impulsivity, that may have interfered with the execution of the task, thereby contributing to some of the observed results. No less important, the results showed an interaction between experimental condition and group, such that the hearing group performed best on the easily nameable stimuli, and this may suggest an impact of language proficiency.

The problem of confounding explicit factors is accompanied by another methodological issue, namely, there is no single agreed-upon task to use when assessing the implicit learning of regularities. The results obtained with different paradigms are not easily comparable with each other, making it difficult to draw clear-cut conclusions. As also pointed out by Hall et al. [25], the observed implicit learning effect is strong with some tasks while it is not statistically detectable with others. The broad variety of experimental tasks might show different degrees of validity for measuring implicit learning. Typically, implicit learning experimental tasks are composed of two integrated phases. In the first phase (familiarisation), participants are exposed to strings of stimuli that contain a covert pattern of regularities, without receiving any explicit information. Then, during the second phase (test), the participant’s implicit learning of those regularities is assessed. If participants’ performance is better (i.e. more accurate or faster, depending on the task) for the familiarised sequences in comparison to unfamiliarised sequences, then the performance is considered to indicate implicit learning as it is assumed participants have been able to discriminate between familiar and unfamiliar stimuli. However, that means that a child with good memory skills could remember and repeat all sequences above chance level, with no differences between familiarised and unfamiliarised stimuli, and consequently obtain a low score in implicit learning, which would make the task not very reliable for its assessment [25].

In addition, to the best of our knowledge, implicit learning has been investigated using patterns of regularities that were based on only one level of complexity. Although this might suit experimental environments, implicit learning in everyday life is most likely based on the ability to process and integrate various levels of complex information. Neuroimaging studies based on an auditory local-global violation paradigm found that 3-month-old infants already process sequences of auditory information at two hierarchical levels, one local (detail-focused) and one global (holistic) [29]. For example, at the very early stages of language acquisition infants’ perceptual system is guided by stimulus-driven attention, so they rely on the processing of salient local (adjacent) elements of speech (e.g. prosodic information such as rhythm or pitch) to segment the speech stream into word units. Yet, within the first months of life, infants become progressively more able to integrate the temporal and hierarchical structure of various linguistic elements and discover the global (non-adjacent) regularities, for example, the non-adjacent rules necessary for morphosyntactic acquisition [30]. The ability to process and integrate information dynamically, at both global and local levels, is necessary in order to process the general structure, make classifications, and form generalizations in different contexts [31].

In this paper, dynamic implicit learning in deaf children with cochlear implants is investigated using a simple reaction time task, namely the Dynamic Temporal Prediction (DTP) task [32]. The DTP is a child-friendly task purposely designed to elicit temporal expectancy of imperative stimuli (also known as the ‘target’, this is the stimulus to which the participant needs to give an immediate response) on the basis of both local and global predictive rules. As described below, in this task children are required to respond as fast as possible to central visual stimuli (cartoon-like characters) preceded by an alerting stimulus (cue). The temporal interval between the alerting stimulus and the imperative stimulus (foreperiod interval) is manipulated by using three different discrete intervals (i.e., short, medium or long). Typically, the higher stimulus predictability for longer intervals results in faster reaction times [33–35]. This effect is commonly known as *foreperiod effect* and is explained by the *hazard rate*, that is the increment in the subjective expectancy that the imperative signal requiring the participant’s response is about to occur [33, 36]. The hazard rate typically increases as the foreperiod increases and is inversely related to the participant’s reaction time. In the DTP task, it refers to a predictive property that changes the participant’s performance within every single trial, at a *local* level [32].

In addition, to explore the adaptation to the task as a function of the *global* predictive rules, we manipulated the statistical distribution of the single foreperiod intervals across the task so that blocks of stimuli could be short-biased, uniform, or long-biased (for details, see the experimental design section). In order to obtain optimal performance in the DTP task, children needed to adjust their motor preparation in terms of response speed. In sum, compared to previous implicit learning tasks that focused on learning the elements that comprise a sequence [20, 21, 23–27], here we were interested in investigating whether deaf children are able to learn the temporal structure of a sequence of imperative stimuli and adjust their motor behaviour accordingly. Based on the results obtained by previous studies involving the DTP task [32], we expect that children who are able to learn implicitly the temporal distribution of the stimuli in each block will perform better than children who are not.

Our aim is to contribute to the discussion about the Auditory Scaffolding Hypothesis and provide complementary evidence regarding the relation between auditory deprivation and the implicit learning of regularities. If there is a link between the domain-general ability to learn environmental regularities and hearing condition as proposed by the Auditory Scaffolding Hypothesis, then deaf children should perform worse than children with typical hearing in any implicit learning task because of their reduced auditory experience. Note that the Auditory Scaffolding Hypothesis refers to both temporal and sequential implicit learning [18]. Previous studies [e.g., 20, 25, 27] focused on the investigation of sequential implicit learning. However, given that oral language is temporally structured, it is possible that this effect could be stronger using a task that is based on the elaboration of temporal regularities, hence our focus. A secondary aim of this study is to propose the DTP as an assessment tool for implicit learning that can avoid confounding explicit factors. Indeed, this task is designed to limit the interference of the explicit processes of working memory and has already been found reliable to elicit implicit learning of global temporal contingencies in children with typical [32] and atypical development (i.e., Down Syndrome; [37]). To avoid verbal recoding and rehearsal, the task requires participants to proactively adjust motor control on the basis of temporal regularities instead of sequences. That means that the participant is asked to detect each item individually instead of small groups (sequences), and this eliminates the possibility of using verbal recoding and rehearsal. In addition, stimuli are temporally distributed and presented in the same location on the screen, which avoids the involvement of visuospatial working memory. Finally, the temporal regularities used in our task are distributed on two levels, local (within-trial) and global (between-blocks), in order to assess the ability of our participants to implicitly process and integrate different levels of predictive information. Specifically, targeting the global predictability allows us to investigate implicit learning at a higher level of complexity, thereby assessing not only the ability to process local regularities, but also the ability to adapt behavioural responses to this implicit knowledge throughout the task. As detailed below, task adaptation is here intended as the ability to proactively adjust response speed to target onset on the basis of different predictive rules that are implicitly instantiated by either local (within-trial expectancy bias) or global (between-block expectancy bias) changes in the inter-stimulus temporal distribution.

## Methods

### Participants

Thirty-five children participated in this study. Seventeen children were profoundly deaf with cochlear implants (age range = 5;04–11;09, mean = 7.96, SD = 1.87) and eighteen had typically hearing levels (age range = 5;02–11;05, mean = 8.05, SD = 1.59). Children were recruited from mainstream and special schools in England. Nonverbal IQ was measured using the Coloured Progressive Matrices [38] and all participants scored within the normal range (i.e., greater than the 25^th^ percentile). The two groups were matched for chronological age, gender, and socio-economic status (geographical area of residence, and parents’ self-reported educational level and profession). Nineteen children (ten deaf) were monolingual native speakers of English, while sixteen children (seven deaf) had English as their dominant language despite being bilingual. The linguistic background of all the participants and the socio-economic status (SES) of their parents is summarised in Table 1 (S1 Table).

**Table 1:** Participants’ linguistic background and parental socioeconomic status.

		deaf	*n/a*	hearing	*n/a*
**spoken language**	English (monolinguals)	10 (58.8%)	–	9 (50%)	–
	English (bilinguals)	7 (41.2%)	–	9 (50%)	–
**SES**^**1**^	unemployed mothers	4 (23.5%)	3	0 (0%)	2
	unemployed fathers	1 (5.9%)	3	1 (5.6%)	3
	mothers with a university degree	11 (64.7%)	3	14 (77.8%)	2
	fathers with a university degree	10 (58.8%)	6	11 (61.1%)	5

*Note*: Data based on voluntary parental reports; Percentages are calculated including the missing data (*n/a*) in the total counting.

^1^socio-economic status.

Inclusion criteria included chronological age between 5 and 11 years old, English as the native and/or dominant language, and no record of cognitive, motor, or sensory impairment (with the exception of hearing loss for the deaf group). Children scoring <25^th^ percentile on a test of non-verbal intelligence (Coloured progressive matrices; [38]) were excluded. Fifteen out of the seventeen deaf children (equivalent to 88%) had a diagnosis of profound bilateral hearing loss (90 dB or greater) with onset by 12 weeks of life. The other two deaf children received the same diagnosis at 96 and 120 weeks, respectively. All children in the deaf group had been fitted with a cochlear implant in at least one ear by the age of four and had been using it for a minimum of three years (S2 Table). Implantation was unilateral for twelve children and bilateral for five. All children were orally educated. Table 2 summarises the characteristics of the two groups. Participation was entirely voluntary. No benefits were offered in exchange for participation; however, all participants received a certificate as a thank you for their contribution. The study was approved by the Ethical Review Board of the 3^rd^ author’s institution (REC 1022, Implicit and Explicit Learning in Children with Hearing Loss).

**Table 2:** Participants’ characteristics.

	deaf (*n* = 17)			hearing (*n* = 18)				
	*M*	*(SD)*	range	*M*	*(SD)*	range	*t*(33)	*p*
**age**	7.96	(1.87)	5;11–11;09	8.05	(1.59)	5;02–11;05	-0.12	0.91
**age at diagnosis**	.30	(.70)	0–2;04	–	–	–	–	–
**age at compensation**^**1**^	1.27	(1.20)	0;02–4;01	–	–	–	–	–
**duration of compensation**^**1**^	6.23	(2.35)	3;06–10;08	–	–	–	–	–
**age at implantation**^2^	1.81	(1.24)	0;02–4;01	–	–	–	–	–
**duration of implantation**^2^	5.70	(2.33)	3;08–10;00	–	–	–	–	–

*Note*: all means are based on age in years.

^1^first hearing compensation either with HA or CI

^2^cochlear implantation.

### Procedure

Potential participants were identified by their teachers, and their families received an information sheet, a consent form, and an optional questionnaire about their linguistic and socio-economic backgrounds. In order to participate, the child’s parents or gatekeepers had to return the consent form completed and signed. Children were asked to confirm their willingness to participate before starting the session. Children were assessed individually by one researcher (the first author). The assessment took place in a quiet room and lasted approximately one hour with breaks provided as needed. The task order was fixed for all participants as follows: 1. Experimental implicit learning task; 2. Digit span (WISC–IV, Wechsler Intelligence Scale for Children–Fourth ed.; [39]); 3. Formulated sentences (CELF–4, Clinical Evaluation Language Fundamentals–Fourth ed.; [40]); 4. Coloured progressive matrices (CPM, [38]). Standardised scores were calculated for the last three tasks.

#### Dynamic Temporal Preparation (DTP) task

**Fig 1:**
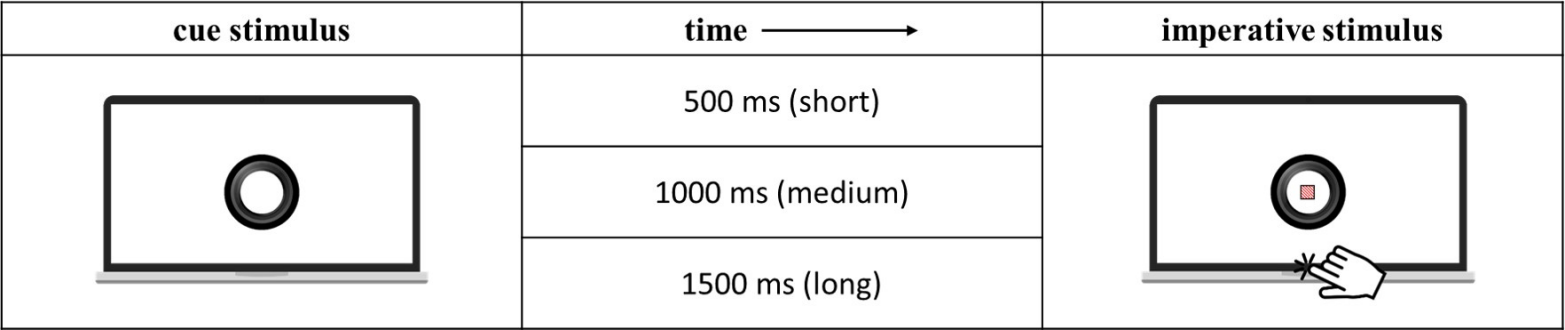
Representation of the DTP task. The visual cue consisted of a black circle representing the lens of a camera. This cue warned children of the upcoming presentation of the imperative stimulus appearing at its centre. The imperative stimulus is here represented with the striped square for illustrative purposes due to copyright restrictions. Participants had to make speeded responses at the target stimulus onset by pressing the laptop spacebar.

*Procedure*. A speeded target detection experimental paradigm, the Dynamic Temporal Preparation (DTP) task [32], was used to assess participants’ behavioural responses (reaction times and accuracy) in order to investigate their ability to implicitly learn and flexibly adapt their responses (i.e., speed and accuracy) to different temporal predictive levels (i.e., local or global) dynamically varied throughout the task. E-prime 2 software (Psychology Software Tools, Pittsburgh, USA) was used to create and administer the task. The data collection apparatus consisted of a Samsung laptop with an Intel® Core™ i7, 4th generation processor, and a 15" IPS Quad HD+ (3200 x 1800) anti-glare screen. Display resolution was set on 1280 × 768. Participants sat comfortably in front of the screen, holding the index finger of their dominant hand on the space bar, and were required to press the space bar as quickly as possible at target-occurrence. Written instructions appeared on the screen and were read out by the experimenter. The text was the following: *“Hi*! *This is a family*! *Here is a dad*, *a mum*, *and their seven children*. *They are playing hide and seek in the woods*. *Your job is to take a photo of them as soon as they appear in view of your camera*. *You can take a photo by pressing the space bar*. *Find them all*! *But take care*, *if you press the bar too soon or too late they will run away*!*”*. No hints were given about the structure of the task. The DTP task structure is illustrated in Fig 1.

Before starting the experimental session, a short training of 60 trials (20 trials per local condition, explained below) was presented in order to ensure participants understood the task instructions. Only during this training did participants receive feedback on their performance at the end of each trial. The feedback depended on the accuracy of their responses, based on reaction times. Specifically, a yellow emoticon with a neutral expression was displayed in case of anticipatory (before target onset) or premature (< 150 ms after target onset) responses; a green smiling emoticon was displayed for reaction times between 150 and 1000 ms; a yellow smiling emoticon was displayed in case of reaction times between 1000 and 1500 ms from the target onset. No feedback was given during the experimental session.

*Experimental design*. Each trial began with the display of a visual cue followed by the presentation of a target stimulus (the imperative stimulus) that remained on the screen for a maximum of 5000 ms. The visual cue consisted of a black circle representing the lens of a camera (total size of the stimulus: 840 × 840 pixels, 144 dpi, 10.62° × 10.54° of visual angle). The target stimulus was displayed centrally within the camera lens and consisted of a picture of one cartoon-like character. The inter-trial-interval was randomly manipulated between 500 and 1,500 ms. Since the visual stimuli and the required response were always the same across the experiment, the only difference between conditions was the level of target predictability.

In the design of this study, the stimulus-onset-asynchrony (SOA) distribution (short, medium, or long) and block-type (short-biased, uniform, or long-biased) were manipulated to investigate the effect of local and global predictive contexts. To investigate the effect of a local predictive context, the SOA between the cue and the target appearance was manipulated. The SOA could vary trial-by-trial within each experimental block in three possible fixed intervals: short (500 ms), medium (1,000 ms), or long (1,500 ms). This manipulation was intended to investigate the local prediction as to the effect of the stimulus hazard rate on task performance.

Probability distribution per each SOA interval in each block was manipulated to investigate the effect of global prediction (global predictive context). Short-biased distribution blocks were biased towards the short SOA, meaning that the probability of having a short SOA was higher in those blocks. Uniform blocks had an even distribution of the three SOAs, meaning that the probability of having short, medium, and long SOAs was equal. Long-biased distribution blocks were biased towards the long SOA and therefore symmetrically opposite to the short-biased blocks. Table 3 shows the probabilities for short, medium, and long SOAs in each global context (S3 Table).

**Table 3:** Simple reaction time task structure.

		Local		
		short (500 ms)	medium (1000 ms)	long (1500 ms)
**Global**	short-biased	60%	32%	8%
	uniform	33%	33%	33%
	long-biased	8%	32%	60%

The task consisted of a total of 9 blocks, three blocks per type (i.e., three short-biased, three uniform, and three long-biased), that were randomly delivered between-subject. Each block included 30 trials, for a total of 270 trials. The experiment lasts approximately 15 minutes. Short resting breaks could be given, if necessary, between blocks. In order to investigate the presence of group differences in relation to the ability to implicitly adjust behavioural performance in terms of speed (reaction times) and accuracy (percentages of not anticipated responses) as a function of either local or global predictive rules, no explicit indication was given to participants about between-block different probabilistic distribution, so that they were not consciously aware of distribution changes. The blocks’ order was randomized between-subjects. This was done in order to avoid spurious effects of bias on performance due to the introduction of local or global fixed predictive contexts.

According to previous literature [33, 35, 37, 41, 42], we expected all children to be able to use the local predictive rule to shape their behavioural performance. Due to the classic foreperiod effect, we expect faster reaction times for long as compared to medium or short foreperiod intervals, regardless of the global properties of the task. Furthermore, if the Auditory Scaffolding Hypothesis [18] is correct we should expect deaf children to exhibit poor implicit learning abilities, thus resulting in a difficulty in the implicit adjustment of motor performance as a function of the block-wise changes in the global stimulus predictability. Alternatively, we should find similar patterns regardless of the auditory condition.

#### Digit span (WISC–IV)

The Digit Span subtest of the WISC–IV [39] was administered to assess verbal working memory skills. This task is composed of two parts (forward and backward). It requires the participant to repeat sequences of digits in the same order (forward task) first, and then in the reverse order (backward task) as they are presented by the examiner. Sequences progressively increase in length (from two to nine digits), and there are two trials of each length. A score of 1 is awarded for every trial correctly repeated by the participant. The task is discontinued if the participant fails to repeat both trials of the same length. For consistency of presentation, a computerised version of the task was used in this study, as recommended by Woods, et al. [43]. Digits from one to nine were pronounced by a native speaker of British English (the 3^rd^ author) and digitally video-recorded, obtaining nine video tracks. The videos included the close-up of the speaker’s full-face to facilitate lip-reading. Each track was then used to compose the standardised sequences and used in the trials at the presentation speed of one digit per second.

#### Formulated sentences (CELF–4)

The Formulated Sentences task (CELF–4, Clinical Evaluation Language Fundamentals–Fourth ed.; [40]) was used as a measure of expressive language and linguistic memory. The participant is asked to formulate complete, semantically and grammatically correct, spoken sentences using given target words (e.g., car, if, because) and contextual constraints imposed by illustrations. The nature of the target words (e.g., nouns, adverbs, adjectives, correlative conjunctions, subordinating conjunctions) stimulates the production of sentences of increasing length and complexity (i.e., simple, compound, and complex sentences). These abilities reflect the capacity to integrate semantic, syntactic, and pragmatic rules and constraints while using working memory. A score of 2 is given for every complete sentence that is semantically and syntactically correct; a score of 1 is given for complete sentences with a correct structure and only one or two deviations in syntax or semantics; a score of 0 is given for incomplete or incorrect sentences. The starting point of the task is based on the chronological age of the participant, and the task is discontinued after a score of 0 in five consecutive trials. For the consistency and clarity of the task delivery, each target word was produced by a native speaker of British English (the 3^rd^ author) and digitally video-recorded. The videos included the close-up of the speaker’s full-face to facilitate lip-reading. The videos at their natural speed were later used to administer the task in this study, following the same procedure as if they had been presented orally.

#### Coloured progressive matrices

Coloured progressive matrices were used to assess participants’ non-verbal intelligence and reasoning ability [38]. The task comprises three sets of 12 items each. The participant is asked to select a missing element from a 3 × 3 matrix in order to complete a pattern. A score of 1 is awarded for every correct trial. All participants scored within the normal range on this task.

## Results

Data were analysed using JASP 0.13.1 [44] and IBM SPSS Statistics 25 [45]. Scores on the coloured progressive matrices [38] were used to control the participants’ non-verbal intelligence. Although all participants’ non-verbal IQ was within the normal range (i.e., greater than 25^th^ percentile), deaf children’s standard scores in the coloured progressive matrices [38] were significantly lower than those of the hearing children *F*(1, 32) = 15.14, *p* < .001, *η*_*p*_^*2*^ = .32. Since the raw scores of the digit span and the formulated sentences tasks are converted to different kinds of standardized scores (scaled and standard scores respectively), in our analysis we used raw scores with age as a covariate. ANOVAs with age covariate were run to investigate the differences between deaf children with cochlear implants and typically hearing participants in verbal working memory (digit span forward and backward) and language (formulated sentences). Table 4 summarizes the results of these analyses (S4 Table). Typically hearing children performed significantly better than deaf children in both forward and backward digit span, indicating greater verbal working memory skills. The formulated sentences scores also differed significantly between the groups. The next analysis compared the performance of the two groups in the dynamic temporal preparation task.

**Table 4:** Standardised tasks means.

	deaf (*n* = 17)		hearing (*n* = 18)				
	*M*	*SD*	*M*	*SD*	*t*(33)	*p*	Cohen’s *d* ^1^
**Digit span forward (raw)**	5.82	2.10	7.89	2.06	2.94	.006	1.00
**Digit span backward (raw)**	4.71	2.54	7.06	1.47	3.37	.002	1.14
**Formulated sentences (scaled)**	5.00	4.33	9.67	3.88	3.36	.002	1.14

Note

^1^Cohen’s *d* is an effect size measure that indicates the standardised difference between two means.

### Data analysis of the dynamic temporal preparation task

Two independent sets of analyses were run to assess children’s performance based on accuracy and reaction times. Group (deaf or hearing), local level (short vs. medium vs. long), and global predictive context (short-biased vs. uniform vs. long-biased) were considered as independent variables. Mean reaction times (milliseconds) and mean accuracy (percentage) were the dependent variables. Omissions, anticipated responses (within the cue and 150 ms after target onset), and delayed responses (1,500 ms after target onset) were considered errors and excluded from the reaction time analysis.

#### Reaction times

Normality assumptions for the nine conditions (3 local × 3 global) were checked using the Shapiro-Wilk test. Results showed a violation of normality for the short condition in the long-biased block for the typically hearing group. These results were due to the presence of two outliers in this group. Removing the two outliers would normalise the data so that assumptions would be respected under all conditions. However, since the violation only appears in one condition, the general results obtained after removing the outliers are consistent with the ones obtained without removing them. Given that excluding two participants would lower the size of our control group from eighteen to sixteen, we decided not to remove them.

**Fig 2:**
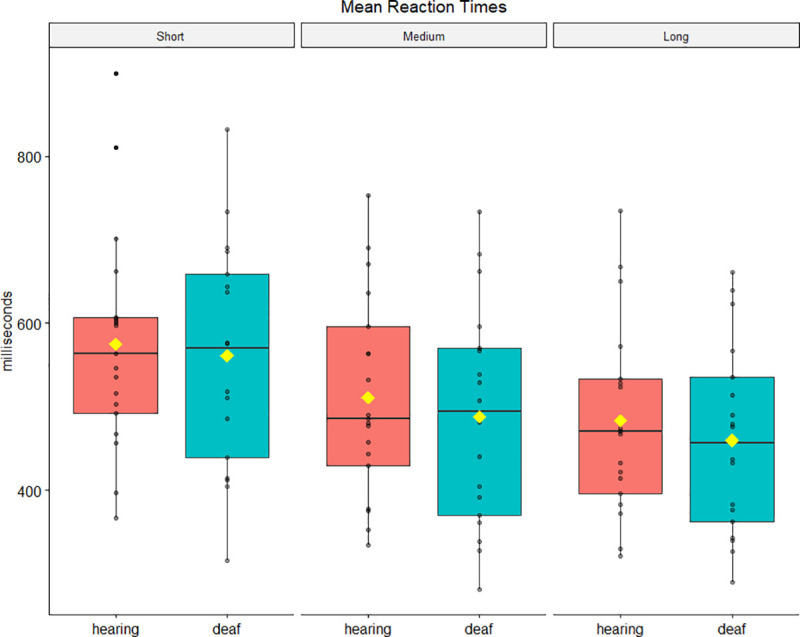
Mean reaction times per SOA condition. Fig 2 was created using the ggpubr package in R [46]. Horizontal lines indicate medians; yellow rhombuses indicate means.

Reaction times data were analysed using a 2 × 3 × 3 mixed ANOVA. Chronological age was included as a covariate. There were no statistically significant differences between group means *F*(1, 32) = .69, *p* = .41, *η*_*p*_^*2*^ = .01. Mauchly’s test indicated that the assumption of sphericity was violated for local predictive context (*χ*^*2*^(2) = 33.37, *p* < .001), and for the interaction between local and global predictive contexts (*χ*^*2*^(9) = 22.37, *p* = .008), therefore the degrees of freedom were corrected using Greenhouse-Geisser estimate (ε = .60 and ε = .76 respectively). A global effect *F*(1.86, 97.34) = 5.92, *p* = .005, *η*_*p*_^*2*^ = .16, and a local effect *F*(1.21, 97.34) = 9.23, *p* = .003, *η*_*p*_^*2*^ = .22 emerged from the analysis. Interactions were non-significant (*p* ≥ .31). Results of the Spearman correlation indicated a significant negative association between age and local predictive contexts, respectively *r*_*s*_ = -.64, *p* < .001 with short SOA, *r*_*s*_ = -.68, *p* < .001 with medium SOA, and *r*_*s*_ = -.69, *p* < .001 with long SOA. No significant correlation was found between reaction times and hearing history (for both duration of amplification and cochlear implant activation) when age was controlled. Fig 2 depicts mean reaction times scores for each condition (at local level) and group. Means and standard deviations are reported in Table 5 (S5 Table).

**Table 5:** Means and standard deviations for reaction times (milliseconds).

		Condition								
		short-biased			uniform			long-biased		
		short	medium	long	short	medium	long	short	medium	long
**Group**	deaf	515.77 (125.98)	436.85 (127.75)	443.09 (116.36)	555.41 (137.59)	487.44 (142.22)	456.65 (120.16)	611.19 (181.30)	536.67 (158.19)	484.61 (143.37)
	hearing	544.79 (132.67)	500.82 (115.88)	461.46 (121.68)	589.44 (124.65)	517.54 (137.29)	496.46 (129.72)	611.58 (173.04)	536.58 (113.30)	505.71 (115.89)

#### Accuracy

As for the reaction times analysis, normality assumptions for the nine conditions (3 local × 3 global) were checked using the Shapiro-Wilk test. Results show a violation of normality for the typically hearing group in all conditions due to a skewness between -2.10 to -0.89, and the violation of normality for the deaf group in three conditions due to a skewness between -1.52 to -0.61, that indicates a ceiling effect for accuracy. Accuracy data were analysed using a 2 × 3 × 3 mixed ANOVA. Chronological age was included as a covariate. There was a marginally significant difference between group means *F*(1, 32) = 4.05, *p* = .05, *η*_*p*_^*2*^ = .11, with deaf children performing worse than hearing children. Mauchly’s test indicated that the assumption of sphericity was violated for local predictive context (*χ*^*2*^(2) = 10.72, *p* = .005), and for the interaction between local and global predictive contexts (*χ*^*2*^(9) = 21.76, *p* = .01), therefore the degrees of freedom were corrected using Greenhouse-Geisser estimate (*ε* = .77 and *ε* = .75 respectively). Neither main global *F*(1.78, 96.28) = 2.05, *p* = .14, *η*_*p*_^*2*^ = .06, nor global x group interaction *F*(1.78, 96.28) = .51, *p* = .58, *η*_*p*_^*2*^ = .02 effects were found, while a significant local predictive context × group interaction emerged *F*(1.55, 96.28) = 6.76, *p* = .005, *η*_*p*_^*2*^ = .18. Other main effects and interactions were non-significant (*p* ≥ .13).

**Fig 3:**
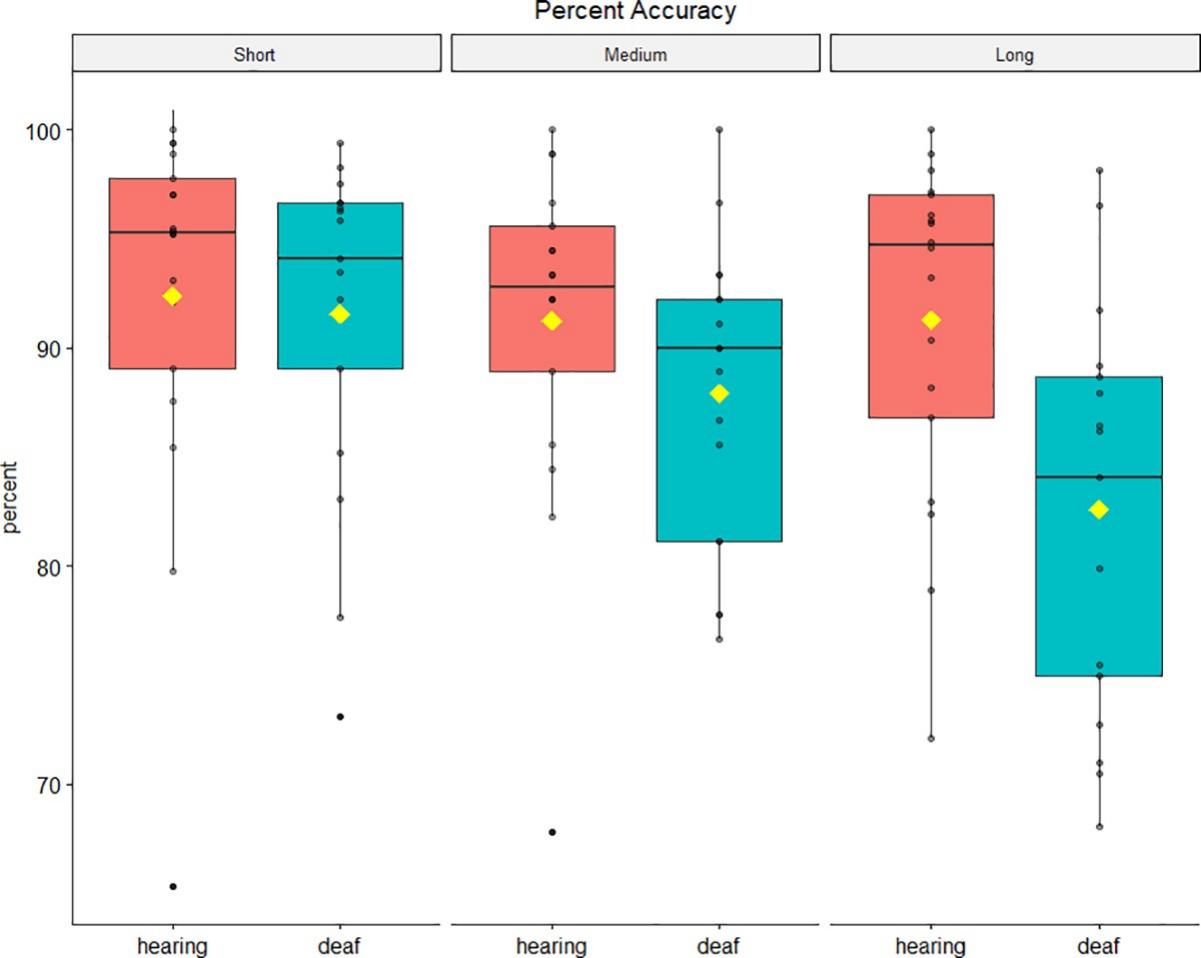
Percent accuracy per SOA condition. Fig 3 was created using the ggpubr package in R [46]. Horizontal lines indicate medians; yellow rhombuses indicate means.

Given that small sample size can increase the risk of a type I error, we conducted post hoc power analyses using G*Power [47, 48] checking whether these results could be due to a lack of statistical power. The results showed that the interaction effect of local predictive context × group reached the statistical power (1 - β) of 0.97, well above Cohen’s criterion for sufficient statistical power [49]. As shown in Fig 3 this interaction was likely driven by the fact that the accuracy scores were not affected by the local probability (i.e., SOA) of stimulus onset for hearing children *t*(17) = .75, *p* = .47, *d* = .18, while performance was significantly impacted by the preparatory interval (lower per cent accuracy for long SOAs) for deaf children *t*(16) = 3.97, *p* = .001, *d* = .96. Fig 3 depicts mean accuracy scores for each condition and group. Means and standard deviations are reported in Table 6 (S6 Table).

**Table 6:** Means and standard deviations for accuracy (i.e. percentage of not anticipated responses).

		Condition								
		short-biased			uniform			long-biased		
		short	medium	long	short	medium	long	short	medium	long
**Group**	deaf	89.76 (8.07)	85.29 (9.58)	81.37 (16.54)	91.77 (7.37)	90.00 (6.66)	82.35 (9.11)	93.14 (11.87)	88.43 (8.43)	83.99 (8.38)
	hearing	92.70 (6.71)	88.52 (9.16)	91.67 (10.31)	91.85 (8.65)	91.67 (9.02)	91.11 (8.93)	92.59 (14.26)	93.52 (7.18)	91.05 (8.62)

To measure the effect of local probability on task performance at the single-subject level, for each child we calculated the delta (Δ) accuracy score as the difference between the mean percent accuracy in short trials minus the mean percent accuracy in long trials. This was done after collapsing all the blocks regardless the global percentage of every single SOA. Then we correlated the single Δ scores with other variables that could potentially impact children’s performance. These included age, duration of hearing compensation and neuropsychological measures. The correlation between Δ accuracy and age was not significant, *r*_*s*_ = .18, *p* = .30. For deaf children, Δ accuracy was also not significantly correlated with the duration of hearing compensation, *r*_*s*_ = .15, *p* = .58 (when chronological age is partialled out), nor with the raw scores of other tasks. Partial correlations between Δ accuracy and other tasks in the deaf group are reported in Table 7 (S7 Table).

**Table 7:** Tasks scores partial correlations for the deaf group.

	Δ accuracy	DS forward	DS backward	FS
Δ accuracy	–			
DS^1^ forward	-.15	–		
DS^1^ backward	-.03	.76**	–	
FS^2^	.20	.45	.53*	–

*Note*: Correlations scores refer to Spearman’s rho. Chronological age is partialled out.

^1^Digit Span (raw)

^2^Formulated Sentences (raw).

* *p* < .05

** *p* < .01

*** *p* < .001

unflagged values are *ns*.

## Discussion

Studies have suggested that the linguistic variability shown by deaf children might result from a deficit in their implicit learning skills [18, 20, 21]. However, several studies have presented data that challenge this hypothesis [23–27], thereby creating a vibrant theoretical and methodological debate in this area of research. The main aim of the present study was to investigate the dynamic implicit learning of temporal regularities in cochlear-implanted deaf children. To the best of our knowledge, this is the first study designed to assess simultaneous implicit learning of temporal regularities on two levels (local and global) in this population. Overall, the similarity of the response speed and accuracy adaptation as a function of global stimulus predictability demonstrates that deaf children with cochlear implants and typically hearing children were comparably able to use the small changes in the presentation rates of the stimuli throughout the task. In other words, both groups were able to implicitly learn the embedded regularities in the stimulus presentation rate, thereby optimising their behaviour by speeding up their responses in the fast blocks and slowing down them in the slow ones. In addition, our correlation analyses showed that this implicit speed adjustment was unrelated to hearing history.

The comparable advantage in response speed and accuracy induced by the changes in the global predictive context overall shows that children were capable of implicit learning. However, worthier of remark, this also suggests that deaf children who participated in our study do not have a deficit in implicit learning of temporal regularities. This is an important aspect of our task, which allows the assessment of implicit learning of temporal regularities without the possible interference of language proficiency, as auditory deprivation is hypothesized, by its very nature, to be strongly associated with reduced experience of processing temporal regularities. Our null hypothesis is confirmed by the results, offering no support to the Auditory Scaffolding Hypothesis [18]. Rather, our results are consistent with the growing body of literature finding no implicit learning deficit in deaf children, thus suggesting that implicit learning is not impaired by a lack of auditory stimulation [23–27]. Given that children with cochlear implants scored significantly behind the typically hearing children in the verbal tasks that involved explicit processing and knowledge (verbal rehearsal and verbal elaboration as measured by the digit span task, and language skills by the formulated sentences task), our results suggest that implicit learning is independent of explicit learning and not significantly affected by poor auditory input.

Strong and significant negative correlations between the reaction times and chronological age suggest that children’s target detection speed (ability to respond fast to the target) increased progressively with age. This result is consistent with findings in the literature that there is a developmental trend in cognitive processing speed that results in faster reaction times over age, during childhood and adolescence [50, 51]. Reaction times were faster in the long SOA condition for both groups if compared with short and medium SOA, probably because of the increased conditional probability of target appearance over time, once the short and the medium SOA limits were passed. This is a classic effect, described in the literature as the “variable foreperiod effect” [33, 34]. Basically, the duration of the intervals between the warning stimulus (cue) and the imperative stimulus (target), as well as the intertrial variability, affect the participant’s state of nonspecific preparation to respond at the moment the imperative stimulus is presented [34], also called the participant’s “response readiness” [52]. A high level of response readiness enables the participant to exert a small effort to reach the level of motor activation required for the successful initiation of a response [33, 52], resulting in faster reaction times. However, even if there was no difference between groups for the reaction times, deaf children’s responses were significantly less accurate in this condition. Adapting the response speed to the task requirements was detrimental for deaf children’s accuracy, while children with typical hearing were able to consistently maintain the same level of accuracy throughout the task. There is consensus that foreperiod tasks strongly tap into inhibitory control [53]. Indeed, a difficulty controlling and inhibiting the response action might explain the deterioration of the performance in long SOA trials, in which the foreperiod effect is more powerful due to the fact that the appearance of the target is perceived as most imminent. Since deaf children showed a stronger foreperiod effect, their inhibitory control seems to be weaker than their typically hearing peers. It is worth noting that this result was not due to poor response readiness in deaf children. In fact, although the difference was not statistically significant, generally faster reaction times were registered in the deaf group compared to the children with typical levels of hearing in each general predictive condition.

This result is not consistent with prior research investigating sequential learning that found slower reaction times in deaf children with cochlear implants compared to children with typical hearing [26]. The discrepancy between our study and Klein and colleagues’ [26] might originate from the different structure of the reaction time tasks that were used to measure implicit learning. While in our study the target was always presented in the same location of the screen and the participant responded by pressing one single key, in Klein et al.’s study [26] the target was presented in one of four possible positions (one of the four screen quadrants) and the participant had to respond by pressing the key corresponding to the target position. Even though the two tasks share some similarities, they require the processing of different kind of regularities: time regularities in the first, and visual-spatial regularities in the second. Both studies concluded that deaf children’s nonverbal sequential learning is comparable to children with typical hearing, however, Klein and colleagues [26] interpreted the slower reaction times in their group of deaf children as indicative of poor sequential processing. The results of the present study do not seem to support this hypothesis. If deaf children with cochlear implants struggle with sequential processing, this seems to be limited to visual-spatial sequences or to emerge with greater information processing demands as in the task used by Klein et al. (2019) [26]. Consistent with our findings, a literature review highlighted that deaf individuals have faster reaction times than typically hearing people in simple detection tasks of visual stimuli [54]. In addition, a previous study investigating the temporal processing ability involved in visuomotor synchronization of adult deaf signers [55] found no deficit in this population.

In sum, our results do not support the hypothesis that experiencing a lack of auditory stimulation at an early age affects either the implicit learning or the processing of sequential temporal regularities. Given the importance of implicit learning of regularities for many aspects of life and linguistic development, it is encouraging that it is independent of hearing status. This also means, however, that the great variability in language outcomes consistently found in many studies with deaf participants cannot be explained by a deficit in implicit learning or processing related to their hearing status, and its causes need to be further investigated. The deaf children with cochlear implants and the children with a typical level of hearing who participated in our study show comparable abilities to implicitly process and learn the regularities embedded on two levels (local and global) in our task. However, our deaf participants show a stronger foreperiod effect than their typically hearing peers, struggling more to control and override the response action as the appearance of the target was perceived as imminent. This behaviour typically depends on executive functioning and suggests a weak inhibitory control [56]. Other studies have found poorer executive functions in deaf children [57], even after controlling for nonverbal intelligence and speed of processing [58]. Our results suggest that executive functions, inhibitory control in particular, could affect performance on the implicit learning test, especially when the task requires the execution of an explicit response. Unfortunately, the design of our study did not include a measurement of inhibitory control, therefore our study cannot provide sufficient evidence to support this hypothesis. This should be further investigated in future studies of nonverbal implicit sequence learning by including an assessment of executive functions, especially inhibitory control. We also suggest that future studies that want to replicate our findings should include more participants. In fact, even though the effect that we found showed a strong statistical power, other effects might not have emerged. The small sample size in combination with the task we used might be underpowered for detecting other hypothetical effects of interest.

## Supporting information

S1 Table Participants’ linguistic background and parental socioeconomic status. (TIF)

S2 Table Participants’ characteristics. (TIF)

S3 Table Simple reaction time task structure. (TIF)

S4 Table Standardised tasks means. (TIF)

S5 Table Means and standard deviations for reaction times (milliseconds). (TIF)

S6 Table Means and standard deviations for accuracy (i.e. percentage of not anticipated responses). (TIF)

S7 Table Tasks scores partial correlations for the deaf group. (TIF)

S1 Data (XLSX)
