# Supplementary figures and images for: Implicit learning of non-verbal regularities by deaf children with cochlear implants: An investigation with a dynamic temporal prediction task
Source: PLoS One. 2021 May 12;16(5):e0251050. doi: 10.1371/journal.pone.0251050 (PMC8115795; doi:10.1371/journal.pone.0251050)

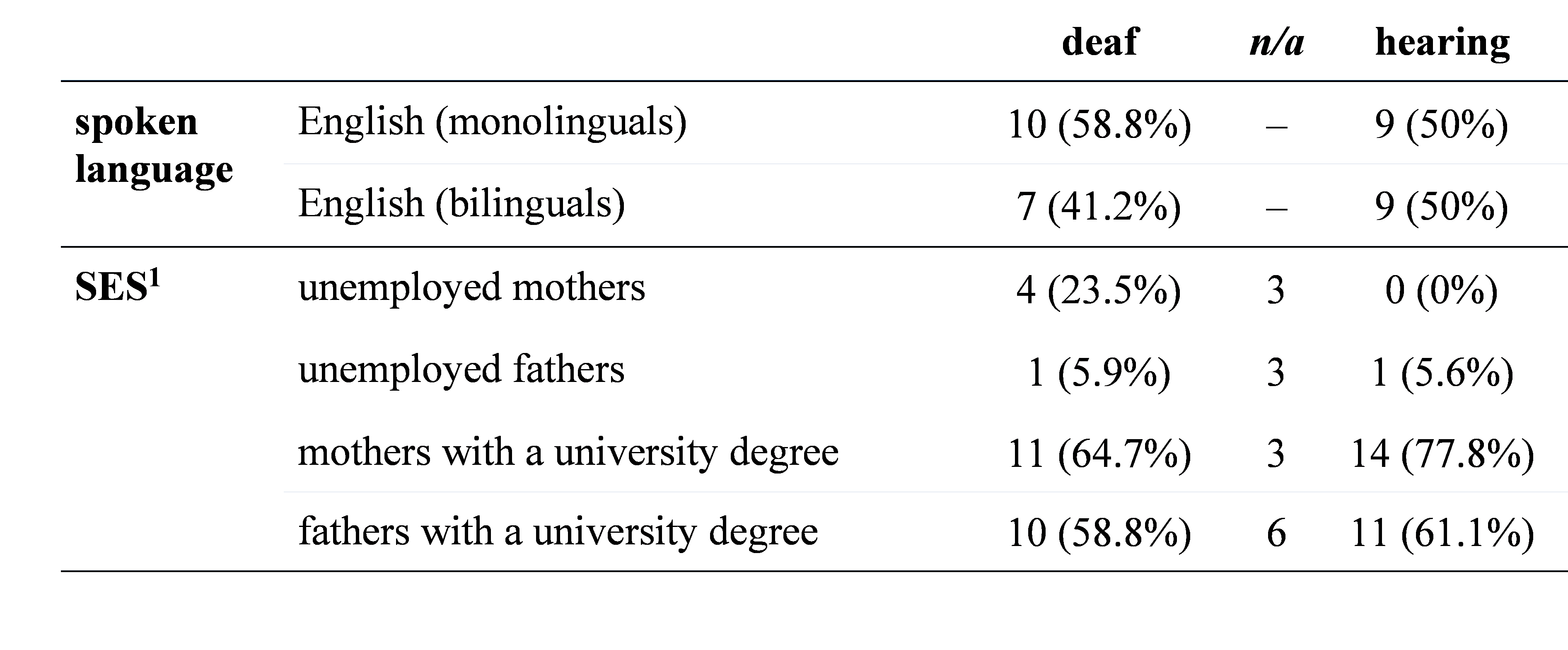

Supplement: S1 Table — (TIF) [file pone.0251050.s001.tif]

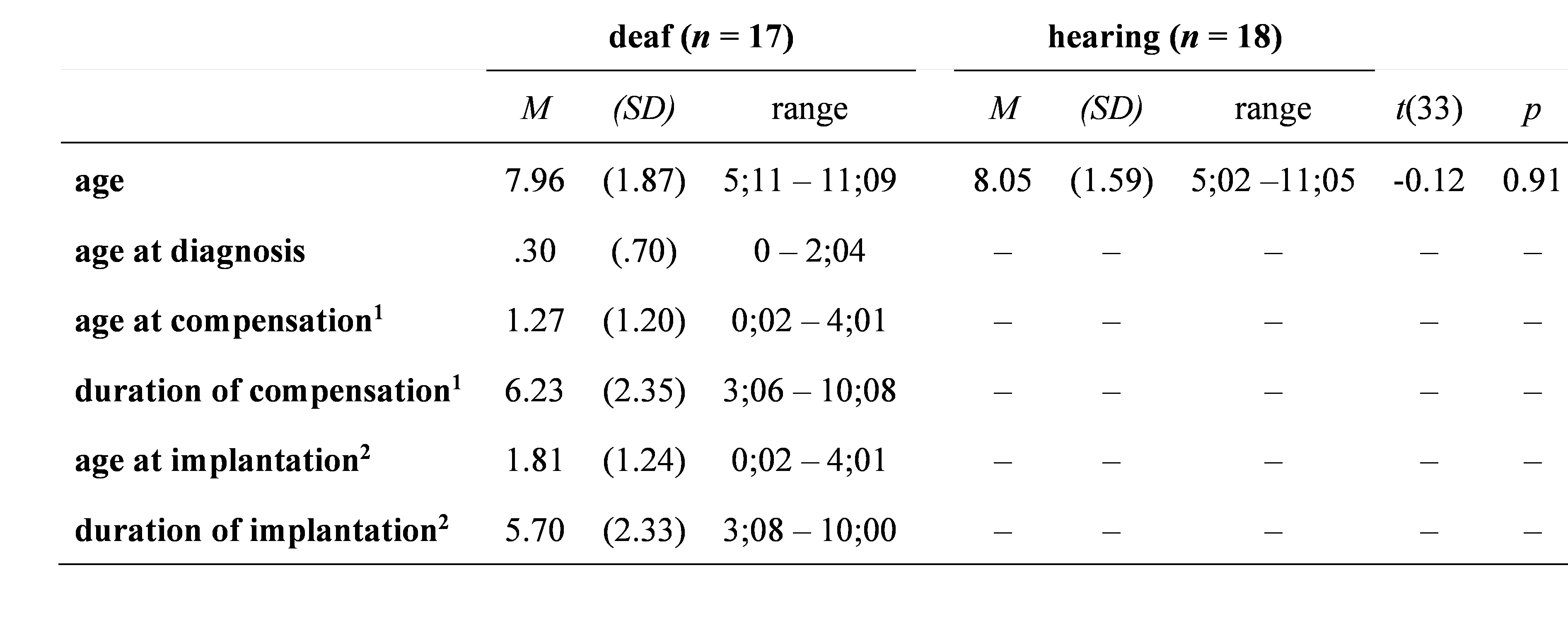

Supplement: S2 Table — (TIF) [file pone.0251050.s002.tif]

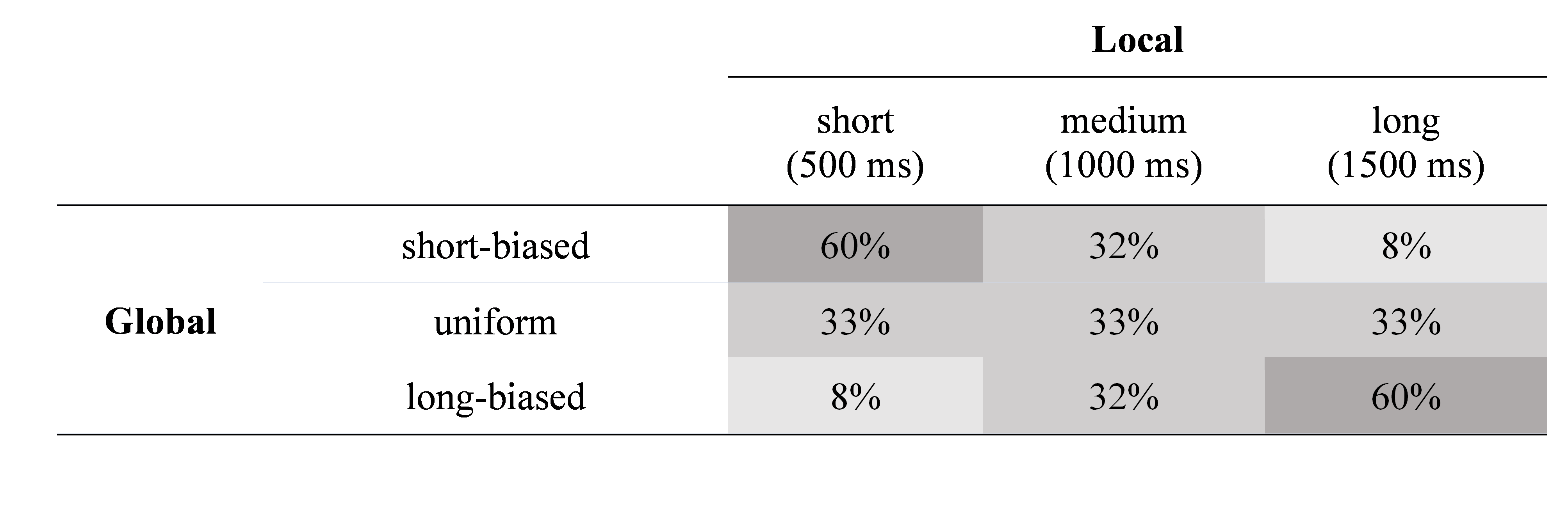

Supplement: S3 Table — (TIF) [file pone.0251050.s003.tif]

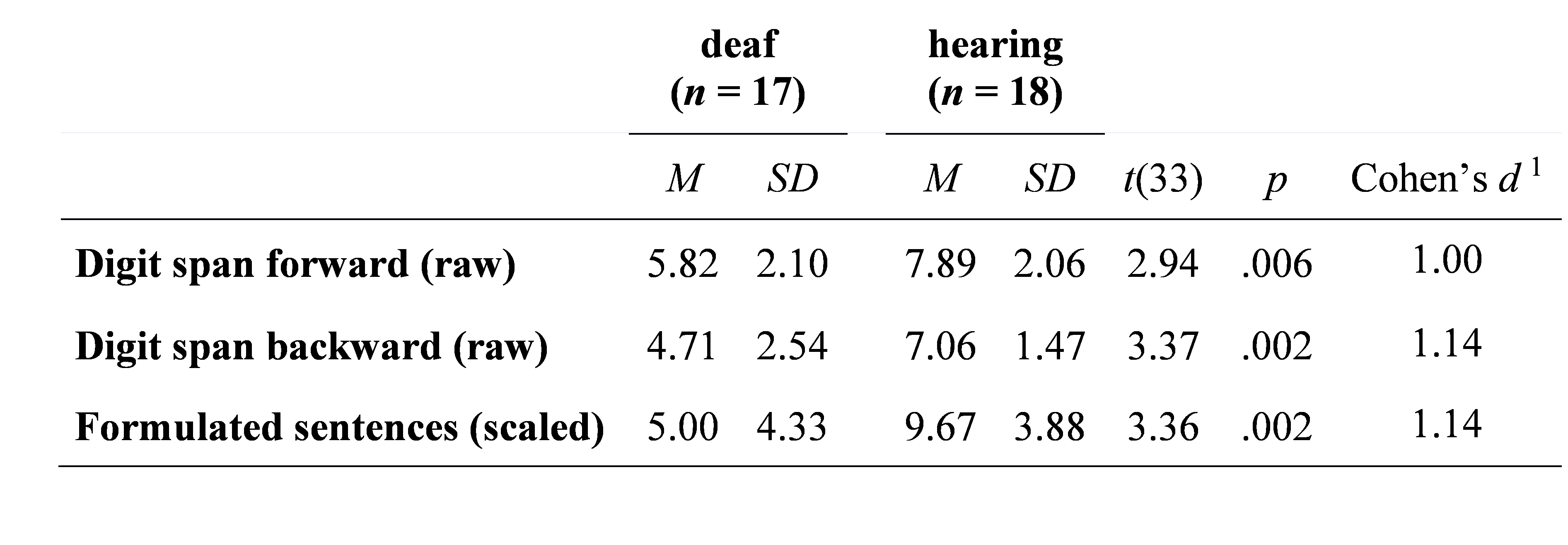

Supplement: S4 Table — (TIF) [file pone.0251050.s004.tif]

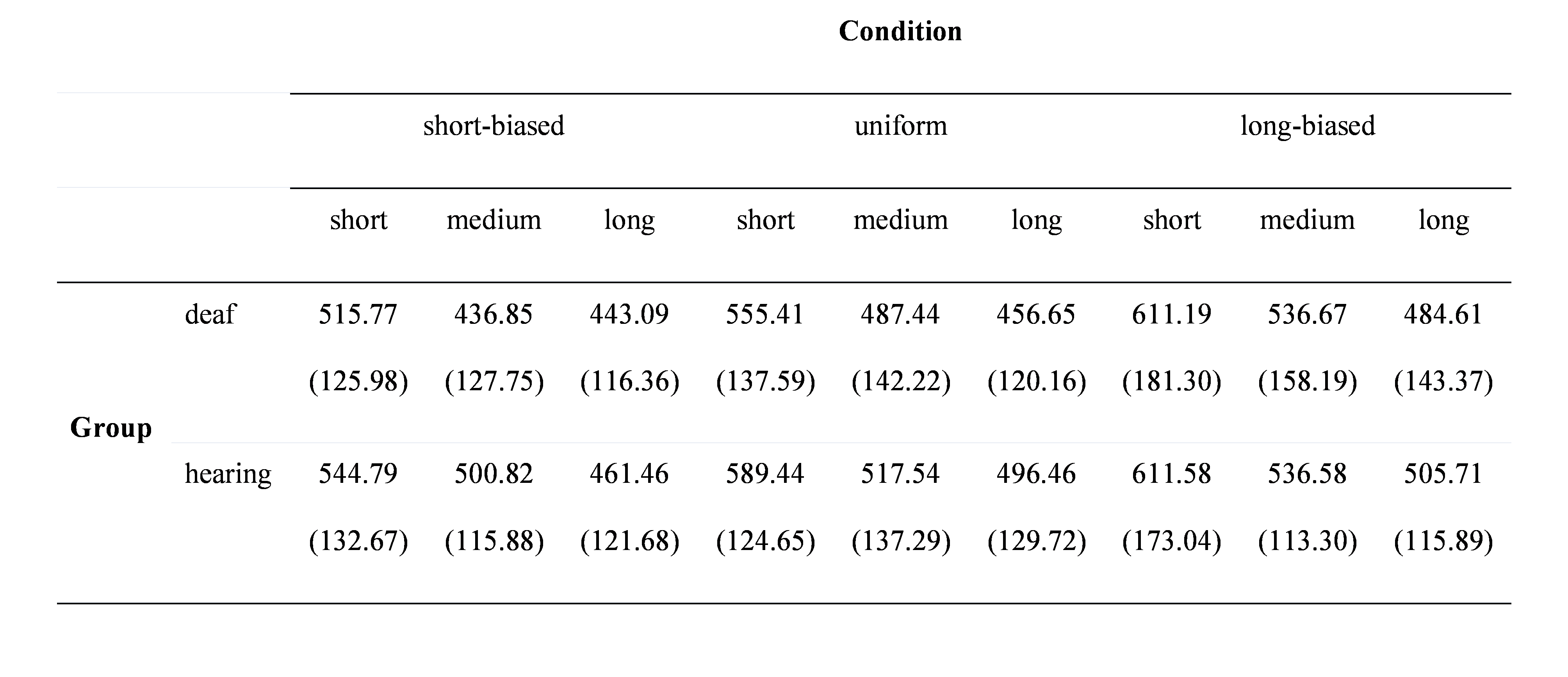

Supplement: S5 Table — (TIF) [file pone.0251050.s005.tif]

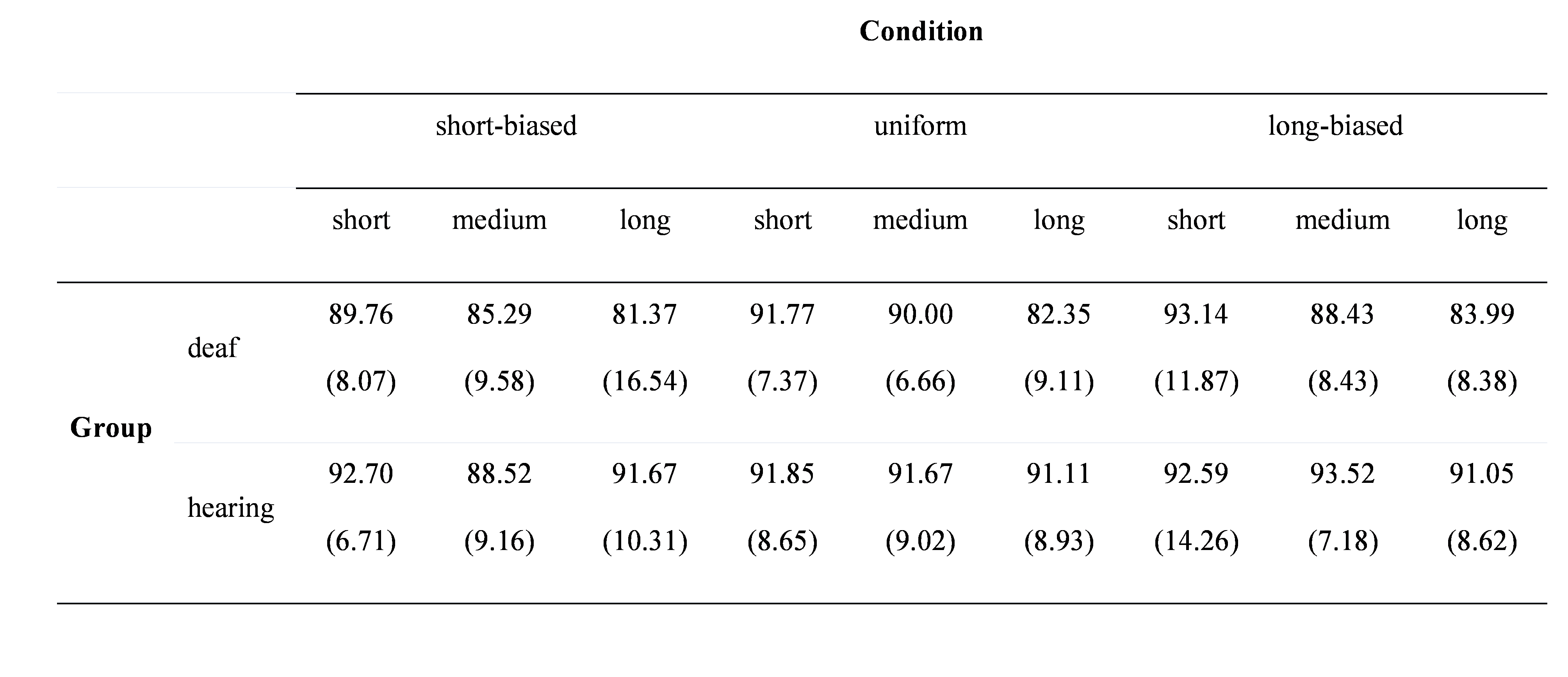

Supplement: S6 Table — (TIF) [file pone.0251050.s006.tif]

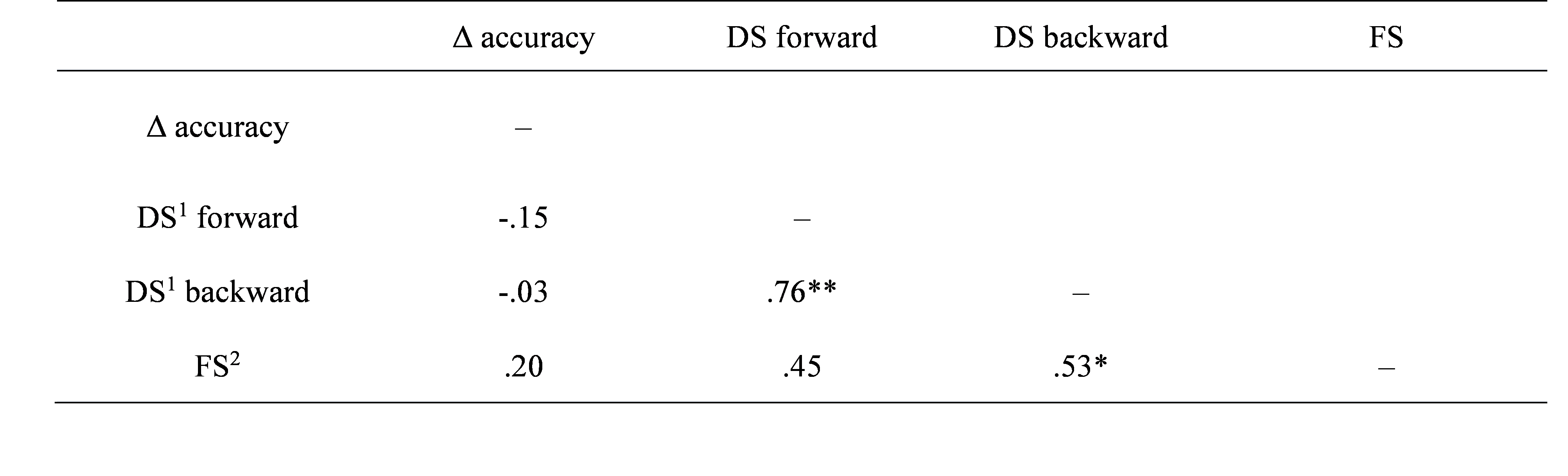

Supplement: S7 Table — (TIF) [file pone.0251050.s007.tif]
